# Role of lupus nephritis classification systems in everyday clinical practice: a questionnaire-based survey of the Renal Pathology Society (RPS)

**DOI:** 10.1093/ckj/sfag028

**Published:** 2026-02-04

**Authors:** Martina Uzzo, Mark Haas, David R W Jayne, Liz Lightstone, Ioannis Parodis, Brad Rovin, Surya V Seshan, Shreeram Akilesh, Agnes B Fogo, Ingeborg Bajema

**Affiliations:** Department of Pathology and Medical Biology, University Medical Center, Groningen, The Netherlands; Department of Pathology and Laboratory Medicine, Cedars-Sinai Medical Center, Los Angeles, CA, USA; Department of Medicine, University of Cambridge, Cambridge, UK; Faculty of Medicine, Imperial College London, London, UK; Division of Rheumatology, Department of Medicine Solna, Karolinska Institutet and Karolinska University Hospital, Stockholm, Sweden; Department of Medicine, Ohio State University Wexner Medical Center, Columbus, OH, USA, Division of Nephrology, Ohio State University, Columbus, OH, USA; Department of Pathology and Laboratory Medicine, Weill Cornell Medicine, New York Presbyterian Hospital, New York, NY, USA; Department of Laboratory Medicine and Pathology, University of Washington, Seattle, WA, USA; Department of Pathology, Microbiology and Immunology, Vanderbilt University Medical Center, Nashville, TN, USA; Department of Pathology and Medical Biology, University Medical Center, Groningen, The Netherlands

**Keywords:** classification, histology, lupus nephritis, survey

## Abstract

**Background:**

Kidney biopsy is the gold standard for lupus nephritis (LN) diagnosis, with the 2018 International Society of Nephrology (ISN)/Renal Pathology Society (RPS) histopathological classification widely used for prognosis and treatment decisions. A survey assessing the use of the 2018 ISN/RPS classification in daily practice was recently conducted on behalf of the RPS.

**Methods:**

An online survey was sent to active RPS members after a webinar that introduced RPS members to the topic. The survey contained multiple choice and open-ended questions and remained open 30 days for completion. Results were analysed anonymously.

**Results:**

Of 562 RPS members, 185 (32.9%) replied to the questionnaire; 180 (97.8%) were pathologists and 120 of these (64.8%) indicated they encounter >20 biopsies with LN per year. The 2018 ISN/RPS classification and the modified National Institutes of Health activity/chronicity indices are used by 92.4% and 88.1% of respondents, respectively. Respondents rated the utility of both systems with a median score of 8 (interquartile range 7–9) on a 1–10 scale. Suggested improvements to the current classification system include greater standardization and simplicity, clearer definitions for grey-zone entities and the introduction of guidelines for new parameters and biomarkers.

**Conclusions:**

This study shows that the 2018 ISN/RPS LN classification is widely used in everyday practice by pathologists. Our results highlight the need for ongoing refinement to facilitate targeted treatment decisions, particularly considering evolving phenotypes and therapeutic advancements in LN.

## GRAPHICAL ABSTRACT

**GRAPHICAL ABSTRACT:**
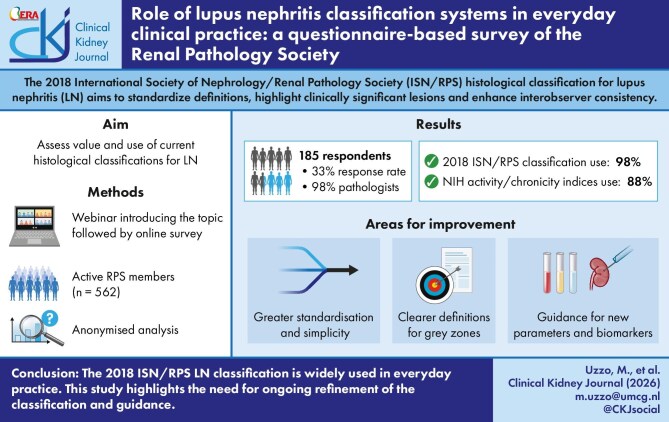

KEY LEARNING POINTS **What was known:**

- Kidney biopsy is the gold standard for lupus nephritis (LN) diagnosis and the 2018 International Society of Nephrology (ISN)/Renal Pathology Society (RPS) histological classification and modified National Institutes of Health (NIH) activity/chronicity indices are widely used for prognosis and treatment decisions.
- Since the late 20th century, LN histopathological classification has been revised multiple times to standardize definitions, highlight key lesions and improve interobserver consistency in line with evolving disease understanding.
- Ongoing therapeutic advancements and biomarker discoveries highlighted the need to refine existing histopathological frameworks for LN: following a Banff-like model, a new RPS committee seeks community input to guide these refinements.

**This study adds:**

- The proposal for a refined LN classification attracted interest from the RPS community, with more than one-third of members completing the survey and providing substantial feedback through open-ended responses.
- This international survey confirms that most kidney pathologists routinely use the 2018 ISN/RPS classification and modified NIH activity/chronicity indices in everyday clinical practice, rating the utility of both systems with a median score of 8 (interquartile range 7–9) on a 1–10 scale.
- Open-ended comments highlighted key shortcomings of current classification systems, emphasizing the need for simplification, standardization and evidence-based updates, along with the inclusion of extraglomerular features, new biomarkers and poorly defined LN-like entities.
- These findings provide critical input for updating the LN classification to better reflect real-world practice, enhancing clarity and clinical relevance.

**Potential impact:**

- A more standardized, evidence-based system may improve daily diagnostic accuracy and communication with clinicians and support personalized treatment strategies.
- The RPS Working Group on LN will incorporate feedback from the international community to assess biopsies from well-characterized cohorts, aiming to refine the classification and support more precise, prognostically driven treatment decisions—an important unmet need.

## INTRODUCTION

Lupus nephritis (LN) is a severe manifestation of systemic lupus erythematosus (SLE) leading to considerable patient morbidity and mortality [1, 2]. Kidney biopsy plays an important role in establishing a diagnosis of LN, together with determination of the histopathological class, which provides important guidance to therapy [3].

Since the second half of the 20th century, the histopathological classification of LN has undergone multiple revisions in order to standardize definitions, highlight clinically significant lesions and enhance interobserver consistency, reflecting advances in the understanding of the pathophysiology of the disease. The International Society of Nephrology (ISN)/Renal Pathology Society (RPS) classification, introduced in 2004 and revised in 2018, represents the last version of this classification system [4–6].

Due to the success of new targeted drugs to treat LN, the discovery of new biomarkers and deeper insights into the pathogenesis of LN, there is ongoing discussion about the need for updating the current ISN/RPS classification [7]. An RPS steering committee involving major experts in the field was established in 2023, with the aim to refine the existing classification system for LN [8–10]. Working in a Banff-like fashion aiming to generate consensus, the committee seeks to engage with the scientific community to gather input on which issues within the classification system should be addressed—and how—to ensure its ongoing refinement and improvement [11].

Within the work scope of the RPS steering committee for LN, an online survey was distributed to the international RPS community to gather insights into the perspectives of kidney pathologists regarding the current histopathological classification system and its practical use in everyday practice.

## MATERIALS AND METHODS

The LN Classification Steering Committee together with the Research Committee of the RPS designed a questionnaire-based survey, powered by the SurveyMonkey online program. The survey included a total of 10 questions: 5 multiple choice questions, with the possibility to add comments and/or justify the answer with open text when appropriate, 3 rating questions (scale from 0 to 10) and 2 open questions. The expected time for completion was ≈10 minutes. Data were collected anonymously. Data from multiple choice and rating questions were automatically converted into accessible graphical representations in Excel (Microsoft, Redmond, WA, USA). Answers to open questions and comments were collected as free text and subcategorized by hand.

A webinar open to all RPS members was held to review the current status of the LN classification and to present the objectives of the survey. The 562 active members of the RPS received a link to the survey by e-mail together with a short description of its aims. The survey remained open for completion for 30 days. The survey’s structure is presented in Table 1: questions 1–3 assessed the respondents’ qualifications and level of expertise in LN, as well as the presence of a multidisciplinary work environment; questions 4–8 focused on the respondents’ perspectives on the practical application and usefulness of the LN classification in real-life settings; and questions 9 and 10 asked the respondents for comments, suggestions and improvements regarding the actual LN classification systems. None of the questions were mandatory. Open-text replies are integrally reported in the supplementary material (Supplementary Tables 1–4).

**Table 1:** Structure of the survey on LN histopathological classification systems in everyday clinical practice, distributed online to the active members of the RPS.

Question	Structure
1. What is your specialty?	Multiple choice: pathologist, researcher, other (please specify)
2. How many biopsies and/or patients with lupus nephritis do you encounter per year?	Multiple choice: <10, 10–20, >20
3. Do you have a (native) renal biopsy meeting at your hospital?	Multiple choice: weekly, monthly, no
4a. Do you use the 2018 version of the ISN/RPS LN classification?	Multiple choice: yes, no, I use the earlier (2004) version, no, I do not use any version of the ISN/RPS or WHO classification
4b. If you do not use the 2018 version of ISN/RPS, why not?	Open text
5a. Do you add scores of activity index and chronicity index to your report?	Yes/no
5b. If you do not add scores of the activity index and chronicity index to your report, why not?	Open text
6. How useful do you feel the 2018 ISN/RPS LN classification is on a scale of 1–10 (with 1 meaning not useful at all and 10 extremely useful)?	Rating question, on a scale from 1 to 10
7. How useful do you feel the activity and chronicity indices are on a scale of 1–10 (with 1 meaning not useful at all and 10 extremely useful)?	Rating question, on a scale from 1 to 10
8. Overall, how well do you think that physicians reading your renal biopsy reports have a clear understanding of what the microscopy report and lupus classification entails on a scale of 1–10 (with 1 meaning no understanding and 10 complete understanding)?	Rating question, on a scale from 1 to 10
9. What do you think could be improved regarding the 2018 version of the ISN/RPS LN classification?	Open text
10. Any other comments?	Open text

Descriptive statistics were performed using Excel. Percentages and medians with interquartile ranges (IQRs) were reported where appropriate.

## RESULTS

**Figure 1:**
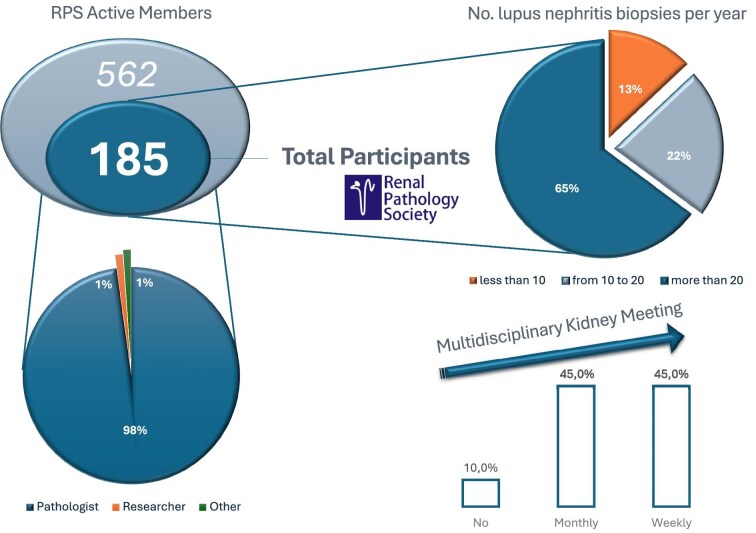
Survey respondents’ details. An online survey was sent between 27 September and 24 October 2023 to the active members of the RPS: 185 of 562 (32.9%) replied to the questionnaire. The first part of the survey assessed the respondent’s qualifications, their level of expertise in nephropathology—particularly in LN—as well as the presence of a multidisciplinary work environment.

A total of 185 of 562 (32.9%) RPS members across the world replied to the online questionnaire. Up to 114 structured replies were collected for a single open-text question (Table 1, Question 9). Among the respondents, 180 of 184 (97.8%) were pathologists, 2 (1.1%) were researchers and the 2 remaining respondents were a nephrologist and a nephrology trainee. The level of expertise in LN was good: 120 of 185 (64.8%) participants encountered >20 biopsies with LN per year, 41 (22.1%) encountered 10–20 and only 24 (13.1%) encountered <10. A total of 165 of 184 (89.7%) respondents regularly discuss kidney biopsy results in a multidisciplinary meeting involving both clinicians and pathologists: 83 (45.1%) on a weekly basis and 82 (44.6%) on a monthly basis. The respondents’ details are reported in Fig. 1.

### Real-life use and evaluation of LN classification systems

**Figure 2:**
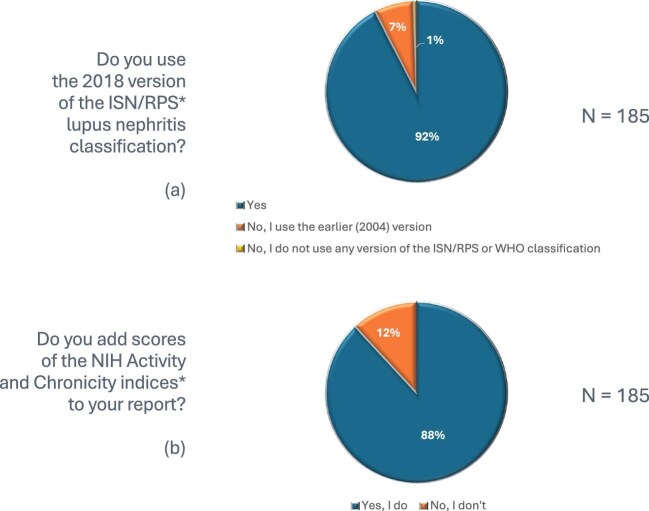
Use of the 2018 ISN/RPS LN classification system [4] and NIH activity and chronicity indices in real-life practice.

**Figure 3:**
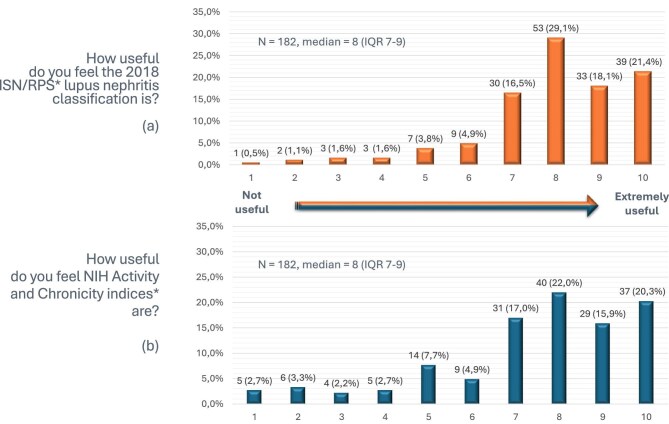
Grading of the utility of the 2018 ISN/RPS LN classification system [4] and NIH activity and chronicity indices in real-life practice.

The 2018 ISN/RPS LN classification [4] is regularly used by the majority of respondents (92.4%), while 13 (7.0%) respondents indicated they prefer the earlier 2004 ISN/RPS LN classification and 1 (0.5%) did not use any LN classification (neither ISN/RPS, nor any of the various updates of the WHO classifications) (Fig. 2a). On a scale of 1 (not useful) to 10 (extremely useful), the median grade of the utility of the 2018 ISN/RPS classification [4] was 8 (IQR 7–9) (Fig. 3a). Those respondents using the 2004 ISN/RPS LN classification [5] justified their choice as follows: (1) the 2004 version is more useful for clinicians, (2) clinicians do not specifically request the 2018 updated edition, (3) both pathologists and clinicians are more familiar with the old version, (4) there was insufficient time to learn about the updated version and (5) the two classification systems are not that different.

The modified National Institutes of Health (NIH) activity and chronicity indices [4] were reported to be included in biopsy reports by 163 of 185 (88.1%) respondents (Fig. 2b). On a scale of 1 (not useful) to 10 (extremely useful), the median grade of the utility of the NIH activity and chronicity indices [4] was 8 (IQR 7–9) (Fig. 3b). Reasons given by respondents for not including the NIH activity and chronicity indices [4] were given as responses to open-ended questions and summarized in the following points: (1) clinicians usually do not ask for NIH activity and chronicity indices [4], (2) clinicians do not use them for making decisions, (3) the extra workload outweighs the perceived clinical benefit, (4) a quantitative evaluation or qualitative description of the degree of activity and chronicity without scoring is less time-consuming and more understandable than the indices (i.e. mild, moderate, severe activity/chronicity or the percentage of glomeruli with active/chronic features), (5) reproducibility is not guaranteed, (6) many biopsies contain <10 glomeruli for evaluation and (7) modified NIH activity and chronicity indices [4] are not evidence-based.

**Figure 4:**
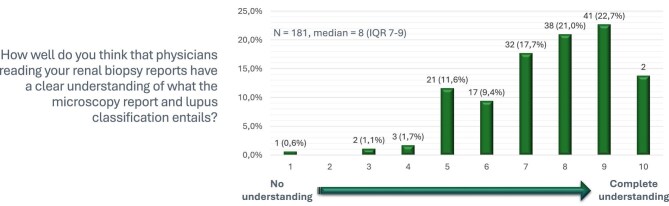
Grading clinicians’ understanding of kidney biopsy reports and lupus classification.

Pathologists rated treating clinicians’ understanding of kidney biopsy reports and lupus classification at light microscopy with a median score of 8 (IQR 7–9) on a scale of 1 (no understanding) to 10 (complete understanding) (Fig. 4).

### Space for improvement

**Figure 5:**
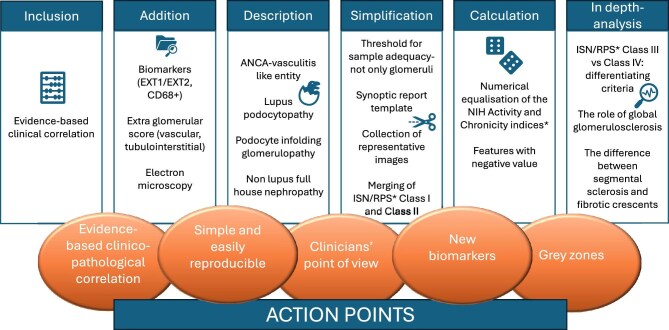
Going towards the ideal histopathological classification system: comments and suggestions.

Suggested improvements of the 2018 ISN/RPS LN classification [4] were as follows: (1) the inclusion of pathological features with evidence-based clinical correlates; (2) the addition of new biomarkers [i.e. exostosin 1 or 2 (EXT1/2) immunostaining for membranous LN, CD68^+^ immunohistochemistry for macrophages to detect endocapillary hypercellularity]; the addition of (3) vascular, (4) tubulo-interstitial lesions or (5), in general, an extraglomerular score; (6) the addition of electron microscopy as part of the score; (7) the attempt to reduce interobserver variability by setting a threshold for sample adequacy for extent of renal cortex—not only for glomeruli—and (8) sharing a synoptic report template; (9) score recalculation through the numerical equalization of the activity and chronicity indices [4] (24 versus 12) or (10) the introduction of features with a negative value (e.g. percentage of normal glomeruli); (11) the description of anti-neutrophil cytoplasmic antibody vasculitis–like necrotizing or proliferative variants and borderline entities such as (12) lupus podocytopathy, (13) podocyte infolding glomerulopathy, (14) non-lupus full house nephropathy; (15) the simplification of existing features (i.e. collection of representative images, merging of class I and II); and (16) in-depth analysis of the criteria differentiating class III and class IV, (17) of the role of global glomerulosclerosis and (18) of the difference between segmental sclerosis and fibrotic crescents (Fig. 5).

Summing up these survey results, the updated classification system should have (1) an evidence-based clinicopathological correlation, (2) it should be simple and (3) easily reproducible; (4) it should encompass the clinicians’ point of view and (5) include new biomarkers and (6) grey zones (Fig. 5).

## DISCUSSION

The goal of this study was to explore the perspectives of experienced kidney pathologists regarding the current LN classification, the modified NIH activity and chronicity indices and the most urgent changes needed [4]. Over time, the LN histopathological classification has undergone multiple refinements and updates in order to standardize definitions, highlight clinically significant lesions and enhance interobserver consistency, reflecting advances in the understanding of LN pathophysiology [5]. The ISN/RPS [4, 5] classification refined the previous WHO system [6] and delineated six distinct classes according to glomerular patterns: class I (minimal mesangial LN), class II (mesangial proliferative LN), class III (focal LN, <50% glomeruli involved), class IV (diffuse LN, ≥50% glomeruli involved), class V (membranous LN) and class VI (advanced sclerosing LN). Moreover, the NIH activity and chronicity indices [12]—first proposed in 1983—were modified to be incorporated into biopsy reports, describing specific glomerular and tubulointerstitial features of active and chronic lesions and grading the individual morphologic components [5].

The ISN/RPS LN classification [4] is an important tool to report different kinds of kidney involvement in LN, providing prognostic information on kidney outcomes and enabling standardized comparisons across studies. The idea of a new, refined LN classification system raised interest in the RPS community: more than one-third of the RPS members completed the survey and they provided a wealth of information on their views of the classification in the open questions. Notably, the question about improving the current classification system received 114 structured responses, including comments and suggestions. A majority of survey participants stated that they use both the 2018 ISN/RPS LN classification and the NIH activity and chronicity indices [4] in everyday clinical practice and graded the utility of both systems with a median of 8 (IQR 7–9) on a scale of 1 (not useful) to 10 (extremely useful). However, open-ended comments calling for improvement underlined the imperfections of current classification systems. Among glomerular diseases, the LN classification is unique in its guidance to therapeutic decisions, as suggested by major international recommendations [3, 13, 14]. However, in contrast with the evidence-based Oxford classification for immunoglobulin A nephropathy [15], the current LN classification system [4] is mainly based on experience, consensus and expert opinion. In the replies to the open-ended questions of our survey, this was a recurrent theme and an urgent action point: the refined classification system should have an evidence-based clinicopathological correlation.

The current LN classification system does not exemplify the pattern of injury and the pathogenic mechanisms underlying individual lesions [7]. A step forward was made when the NIH activity and chronicity indices [12] were modified and introduced for all classes in 2018 [4] to improve the prognostic value of kidney biopsy; the chronicity index and its components—but not the activity index—were significantly associated with long-term impairment of kidney function in recent studies [16, 17]. In this context, new efforts have led to the identification of different disease phenotypes based on pathogenic pathways [18]. In 2022 Bolognesi *et al.* [19] analysed kidney biopsy data from class III/IV ± V ISN/RPS LN patients from the BLISS-LN trial (NCT01639339) [9] to identify new histological patterns beyond the traditional classification. Using cluster analysis, the study identified two main subgroups—membranoproliferative-like (MPGN-like) and vasculitis-like—which could provide a more pathogenesis-oriented classification and improve treatment strategies. More recently, Danaher *et al.* [20] utilized single-cell resolution spatial transcriptomics to study the cellular composition, phenotypes and spatial localization of cells in paediatric LN biopsies. Using a 960-gene panel, they discovered that even histologically normal-appearing glomeruli within the same patient’s biopsy may nevertheless harbour gene expression signatures of inflammation and injury. Integrating molecular methods with traditional histologic classification schemes will undoubtedly increase our understanding of LN pathogenesis and heterogeneity. Informative biomarkers that are identified from those studies may be deployed as traditional tests (i.e. immunohistochemistry) in laboratories without access to spatial transcriptomics platforms [21]. For now, these findings will need further study and validation before they can be incorporated into future updates of the LN classification—but they are reflecting exciting new developments that will ultimately lead to transformation of the LN classification system with the incorporation of new techniques in addition to the traditional use of light microscopy, immunofluorescence/immunohistochemistry and electron microscopy.

An important suggestion that came out of the survey was that the following entities should be described and included in the classification system: lupus podocytopathy, podocyte infolding glomerulopathy and non-lupus full-house nephropathy. Also, the designation of a vasculitis-like entity as a separate class was a recurring suggestion. It is clear that an ideal, future classification system should encompass all those rare, exceptional entities with sometimes unclear boundaries because of the lack of proper definitions and nomenclature. In particular for non-lupus full-house nephropathy, the boundary of a patient not having a diagnosis of SLE is crossed: the group feels that currently LN should only be diagnosed and classified in patients with a diagnosis of SLE.

It has been known for some time that different histopathologic patterns observed in light microscopy, immunofluorescence and electron microscopy may indicate distinct immunologic pathophysiologic mechanisms. Consequently, emerging targeted treatments could be more easily tailored to these specific mechanisms [8–10]. Moreover, in the context of precision medicine, emerging data have revealed molecular underpinnings of LN pathology that compliment histological classifications. Consequently, a subset of potential biomarkers was suggested to be included in the LN classification system: EXT1 and EXT2 for membranous nephropathy associated with autoimmune diseases [22], as well as glomerular CD68^+^ cells as a surrogate marker for endocapillary hypercellularity [23] having prognostic and/or therapeutic significance. Whereas the ISN/RPS LN [4] classification currently provides some clues to guide the choice of the available regimens—which mostly have a broad and non-specific effect on the immune system—it would be extremely helpful if a future update provides indications tailored to newly developed targeted therapies.

Another crucial aspect missing from the current LN classification is the impact of the disease in the extraglomerular compartments, including tubulo-interstitium and blood vessels. Despite the incorporation of the modified NIH activity and chronicity indices [4], the exclusion of these compartments from the main classification was viewed negatively by the survey population. To address this gap, the introduction of an extraglomerular score was proposed. On the one hand, all these additional aspects will provide a clearer picture of individual patients and their disease phenotype; on the other hand, they will add complexity to the classification. According to a significant proportion of the survey population, the lack of standardisation and simplicity are unmet needs in the current LN classification. Indeed, previous studies have shown poor interpathologist agreement in scoring kidney biopsies using the ISN/RPS classification system and the NIH activity and chronicity indices, although this agreement improves with experience [4, 24, 25]. In this context, many suggestions were proposed in this survey to enhance reproducibility, including the distribution of a synoptic report template for writing reports, an official collection of representative images showing specific kidney lesions and the establishment of a threshold for sample adequacy in the extraglomerular compartment.

This study has limitations. Although the survey was distributed to RPS members worldwide, no demographic data were collected due to the required anonymity of the respondents, thereby impeding subset analyses by geographic region. Additionally, since the overwhelming majority of respondents were pathologists, the findings may not reflect clinicians’ perspectives and should be interpreted with caution. This survey may be regarded as a starting point aimed at renovation of the LN classification from the perspective of those who are responsible for scoring kidney biopsies first. Moreover, the survey was intentionally kept concise to maximize the response rate and to ensure a high completion rate: this shortness limited the evaluation of some important topics.

Future follow-up studies involving different kinds of clinicians (i.e. nephrologist but also rheumatologists and immunologists) are planned to provide a more comprehensive, multidisciplinary understanding of classification utility and implementation challenges.

In conclusion, this study shows that the latest 2018 ISN/RPS LN classification [4] is widely used by pathologists in everyday practice. The comments collected from the RPS members acknowledge and encourage the need for ongoing refinement to facilitate targeted treatment decisions, particularly considering evolving phenotypes and therapeutic advancements in LN. The ideal classification system should be simple, easily reproducible and evidence-based—with clear relevance to prognosis and treatment indications—and should encompass both the pathologist’s and the clinician’s point of view. The introduction of new biomarkers and grey-zone entities is strongly suggested.

The RPS Working Group of LN classification will consider the ideas and suggestions of the international community to evaluate biopsies from extensively phenotyped cohorts, to modify the classification and to facilitate improved targeted treatment decisions based on prognostic pathologic findings—a current unmet need.

## Supplementary Material

sfag028_Supplemental_File

## Data Availability

The data underlying this article will be shared upon reasonable request to the corresponding author.
