# Supplementary material for: Role of lupus nephritis classification systems in everyday clinical practice: a questionnaire-based survey of the Renal Pathology Society (RPS)
Source: Clin Kidney J. 2026 Feb 4;19(3):sfag028. doi: 10.1093/ckj/sfag028 (PMC13006876; doi:10.1093/ckj/sfag028)
Supplement: sfag028_Supplemental_File [file sfag028_supplemental_file.pdf]

Supplemental material

Table of contents:

We here enlist all the original answers that respondents gave to the open text questions of this survey. Answers are reported in the order of submission.

1. **Supplemental Table 1 - Respondents' answers to Question 4b:**
   'If you do not use the 2018 version of ISN/RPS, why not?'

2. **Supplemental Table 2 - Respondents' answers to Question 5b:**
   'If you do not add scores of the NIH activity index and chronicity index to your report, why not?'

3. **Supplemental Table 3 - Respondents' answers to Question 9:**
   'What do you think could be improved regarding the 2018 version of the ISN/RPS lupus nephritis classification?'

4. **Supplemental Table 4 - Respondents' answers to Question 10:**
   'Any other comments?'

Supplemental Table 1 - Respondents' answers to Question 4b (open text): 'If you do not use the 2018 version of ISN/RPS*, why not?'.

**Bajema et al., Kidney Int, 2018*

ISN/RPS, International Society of Nephrology/Renal Pathology Society

	**If you do not use the 2018 version of ISN/RPS*, why not?**
1	I use the 2004 ISN/RPS classification, because, it seems to me, to give more useful information for clinicians.
2	Not fundamentally new and helpful; lesions such as vasculitis still not considered; too little emphasis on tub-int changes
3	It's not that different and both I and my clinicians are familiar with the 2004 version
4	Used to the earlier version
5	I use all the language of the 2018 version, but do not report as NIH indices.
6	Our nephrologists are not acquainted with 2018 classification.
7	Infrequently read medical kidney biopsies - only for preliminary diagnosis
8	I was trained on the earlier version and have not taken the time to become fully familiar with the 2018 changes
9	I finished fellowship before the new classification
10	I have not yet adapted the new one
11	I report both WHO and 2018 ISN classification in diagnosis
12	Not requested by nephrologists

Supplemental Table 2 - Respondents' answers to Question 5b (open text): *'If you do not add scores of activity index and chronicity index* to your report, why not?'.*

**Bajema et al., Kidney Int, 2018*

ISN/RPS, International Society of Nephrology/Renal Pathology Society

	**If you do not add scores of activity index and chronicity index* to your report, why not?**
1	Physicians do not use it for making decision.
2	The Nephrologists do not ask for them; we give qualitative, descriptive data.
3	Will apply it soon
4	I discuss qualitatively the degree of chronicity with clincians and include it in my report but don't quantitatively score it
5	Not reproducible, as you know
6	Too tedious. I give an overall % or proportion of glomeruli with active lesions and those with chronic lesions.
7	I report them as percentages in the microscopic description, and the indices could be assigned with the info i provide in the report. Our clinicians seem very comfortable with the way these are reported. I have a couple reasons why I do not translate these into NIH activity and chronicity indices:<br>1. Older literature from when these NIH indices were previously used highlighted multiple flaws with them. It feels like a research tool implemented into clinical reports without being clinically validated first.<br>2. The variables at the suggested cutpoints have not been shown to be reproducible among pathologists<br>3. The variables at the suggested cutpoints have also not been shown to correlate with clinical presenting variables, response to therapy, or overall prognosis. They have not been demonstrated to be clinically meaningful at the suggested thresholds, and this is the main reason I do not use them.<br>4. From a working standpoint, the scores sometimes seem to underestimate the severity of activity. My comments instead give the precise % of glomeruli involved by crescents and other specific lesions, which I prefer over translating this it into a number that may undersell activity.<br>5. Tubulointerstitial and vascular lesions not well-accounted for.<br>6. As a minor issue, from a biologic standpoint, I struggle with the notion of assigning points separately to necrosis and crescents, as one leads to the other. However I recognize that this may be a way of adding more activity points for more cellular crescents compared with fibrocellular ones.
8	Do not believe they are reprodicible, so have only used when comparing biospies from the same patient when scored on the same day by the same pathologist.
9	Too much extra work in relation to the impact of the indices on the handling of the patient; the clinicians do not ask for them.
10	Useless for clinicians. Very difficult for them to understand
11	The clinicians are not using it for management
12	Only provide preliminary results
13	I'm a nephrologist, my pathologist does not add it
14	The nephrologists/rheumatologists have no need for it - they interpret the activity from the report.
15	I state which lesions are present but do not use the index as it is not evidence-based.
16	See above
17	No by number, I do it in comments and in the meeting
18	I classify as mild, moderate, severe without numerical value
19	No need from nephrologist<br>This pathologist sees no data to support the utility of this scoring, which add more work
20	Not requested by nephrologists
21	Many of my biopsies contain <10 glomeruli for evaluation

Supplemental Table 3 - Respondents' answers to Question 9 (open text): *'What do you think could be improved regarding the 2018 version of the ISN/RPS* lupus nephritis classification?'.*

**Bajema et al., Kidney Int, 2018*

ISN/RPS, International Society of Nephrology/Renal Pathology Society

	What do you think could be improved regarding the 2018 version of the ISN/RPS* lupus nephritis classification?
1	More information about vascular lesions. More granularity in the stratification of active/chronic for class III and IV.
2	Should the total number of glomeruli (as denominator in computing for the activity and chronicity scores) include those from the IF? Lesions aren't readily visible on IF and would make the scores lower than they actually are.
3	I belive that the subclassification of global and segmental lesions (A or C) has not been studied properly.
4	clarifying the role of sampling in describing tubulointerstitial inflammation, tubular atrophy and interstitial fibrosis, perhaps by setting thresholds for sample adequacy.
5	Clarify how double contours fit in the classification in the absence of TMA.<br>Please make a new table for diagnostic categories like Table 3 of in Weening, KI Vol 65. 2004.
6	Inclusion of vascular changes (e.g. vasculopathy, arteritis, hyalinosis, arteriosclerosis etc
7	Better definition of classes
8	Too cumbersome. Activity is not easy to calculate and will differ pathologist to pathologist. Inter observer variability.
9	terminus mesangial proliferation vs hypercellularity
10	It would be helpful if the criteria for class III and IV are more clearly defined.
11	FINALLY also incorporate vasculitis! Place more emphasis on tubulo-interstitial lesions including place more significance on active inflammation and chronic lesions.
12	It bothers me that when you have 1 globally sclerosed glomerulus and minimal (<5%) IFTA, you immediately get 3/12 points in chronicity index
13	I think it is fairly clear
14	to add scoring systems of the tubulointerstitial injury and vascular lesion
15	CLINICIANS' ADEQUATE UNDERSTANDING OF HISTOPATHOLOGICAL FINDINGS. AND THE EFFECTS OF IMMUNOSUPPRESSION
16	I think global and segmental glomerulosclerosis should be classified and scored separately. I'm not sure interstital inflammation belongs in the "activity" scoring systemn since inflammation is often concentrated in areas of chronic injury (i.e. IF/TA). My clinicians manage cellular crescents and *especially* fibrinoid necrosis differently from other markers of activity so reporting needs to call these (and for that matter, all the other factors) out separately -- which makes for a complex and wordy report. It would be helpful to know whether doing all the extra labor of scoring each individual thing and calculating a score really has any correlation to clinical status. It seems like a lot of extra work.
17	% "normal;" (i.e. no light microscopic pathologic lesion) has shown predictive value in ANCA-GN. Perhaps this could be added with a negative value to be subtracted from the total activity score. For example, >50% -3, 25%–50% -2, <25% -1.
18	While the specific details can be important for research purposes, it is very time consuming for pathologists and I do not think the nephrologists are looking at things in so much detail or treating based on those specifics.
19	Just add some explanation about activity index of membranous lupus nephritis
20	More tinkering will make little difference
21	To include vascular and tubulointerstitial injury.
22	the definition and/or cut off point to name combination class for class V and other class
23	Harmonization of terminology with the RPS consensus definition

24	To improve activity and chronicity index
25	The classification could include tubulo-interstitial and vascular lesions (with differentiation between chronic and active lesions)
26	the Class IV-C especially when there are many obsolete glomeruli but not reaching the threshold for Class VI is confusing
27	The 2018 classification serves glomeruli very well - but essentially ignores vessels and the tubulointerstitium
28	Make the definition more clear for each parameter with a representative image
29	I still do not understand the basis for not classifying a lupus nephritis class V rather than II+V when detecting mesangial immune deposits.
30	Demonstrate that the NIH activity and chronicity indices are associated with clinical variables, similar to the IgAN classification process, or similar to AJCC staging in the neoplastic world.<br>Otherwise, what is the purpose of translating our words into numbers, if those newly generated variables have not been shown to add meaning?
31	definition of class V include endotheliaitis/arteritis in activity index
32	Clarification on the significance of global glomerulosclerosis and some guidelines for reporting lupus podocytopathy
33	An update on the activity and chronicity index thresholds with regard to clinical application, e.g., does an activity score of n
34	extra glomerular lesion classification
35	Better definition for endocapillary hypercellularity.<br>Addition of CD68 IHC to assess endocapillary hypercellularity.
36	Addition of more detailed tubule, interstitial and vascular scoring
37	Formal publication on the new update as a table with detail microscopy on each entity
38	Include a detailed assessment of tubulointerstitial and vascular lesions
39	tubular injury, vascular lesion, podocyte injury
40	The chronicity index includes glomerular segmental sclerosis separately from fibrous crescents. It is not always so clear cut to separate those two. Although I understand the rationale behind it, I still think it could be improve. Interstitial nephritis/TBM and interstitial deposits should be mentioned somewhere in the classification. At the moment, only the interstitial inflammation is part of the activity index. Same for vessels. We all add it somewhere in the diagnosis/comments/microscopy but it is definitely missing. Maybe we need a glomerular score, and an additonal score?
41	Total activity and chronicity score do not have the same scale bar. Often, quite active lupus nephritis IV with minimal chronicity will have activity score like 12 or 13 out of 24 and chronicity 3 or 4 out of 12 (chronicity being IFTA 5% and one globally sclerosed glomerulus and one or no fibrous crescents), so 3 or 4 out of 12 looks exaggerated.
42	This revised version of ISN/RPS lupus nephritis classifications better than the previous versions.<br>However, clinical validation studies may further identify and improve the prognostic implications of the pathologic parameters as well as refine the activity and chronicity indices and include the prognostic importance of the various vascular lesions.
43	Simplify further
44	evidence based
45	Simplify to a minimum to improve understanding and relevance.<br>e.g. do we need to distinguish I and II? do we need to distinguish III and IV? why not just mainly mesangial/endocap prol/mainly membranous. Guidance on reporting podocytopathy, TI and vascular lesions Guidance on reporting TMA/antiPL: often over-looked
46	The current classification and activity index don't include many significant findings, such as TMA, vasculitis, and interstitial nephritis. Many nephrologists tragically don't treat patients with 0 activity but chronicity, despite the presence of TMA, immunocomplex deposits that are mesangial and intermembranous with proteinuria yet not sufficient to classify as class V. Much nuance is lost in the current classification.
47	Factor in podocytopathic changes in lupus classification (I&II) and when to state this clearly as opposed to overlapping changes due to NSAIDs
48	vasculitis and interstitial infiltrate

49	I think this explains better the lupus activity/state.
50	The activity and chronicity index should be integrated into the classification and standardized for class III and IV lupus nephritis. Although I report the index in my comment, I am not sure that they are using the parameters in managing the patient.
51	better specify extension and type of IFTA inflammation.
52	Good correlation should be made between clinical value, reliability of the marker and efficiency aspects. Often, all these grading schemes are time-consuming and the clinicians do not even use them or need them (eg activity scores)
53	Am eagerly anticipating the next version of the ISN/RPS classification system that will hopefully include APLAS and propose criteria for lupus podocytopathy.
54	Vascular disease
55	add specific LN lesions such as podocytopathy, TMA and PIG with specific evaluation items
56	activity index
57	-Clarification regrading minimal criteria for low grade/segmental membranous features with no class III or IV features. What degree of membranous nephropathy alone is enough to get to class V. (i.e, few subepithelial deposits by EM? at least segmental MN features by LM, no LM features but segmental membranous pattern IF staining, etc.?).<br>-Similarly, the immune complex burden in pure class V cases is variable even when diffuse and global; some sort of scoring/activity/chronicity index system within class V might be helpful both for cases with very abundant deposits as well as cases with all resorbed deposits (i.e., stage IV MN) for which class V directed treatment may not be helpful<br>- A consensus recommendation or discussion regarding standard use of EXT1/2 immunostaing in lupus nephritis; should this be standard practice as data coming out suggest it is a good prognostic biomarker?<br>-Additional recommendations regarding the diagnostic criteria for lupus nephritis, specially recommendation regarding diagnostic reporting when patient's do not meet ACR criteria for lupus or we don't have all the serologies back at time of biopsy. Similarly at least a comment regarding ISN/RPS viewpoint on ACR vs SLICC criteria for lupus. Should we be using the diagnostic terminology "non-lupus full house nephropathy" or should we just top line as immune complex GN with a comment regarding ddx.<br>-Recommendation for classification of cases that are class II or V by light microscopy but more pronounced subendothelial are seen by EM than expected.<br>-Clarification regarding the interstitial inflammation component of the activity index when other class III or IV features are not present. For example in a class V lupus nephritis with some interstitial inflammation would we provide an activity score? Or the glomerular changes are entirely chronic and there is some interstitial inflammation; is the lupus nephritis considered active?<br>-Clarification regarding scoring interstitial inflammation in scarred versus non-scarred cortex<br>-Consideration of inclusions or arteriosclerosis and arteriolar hyalinosis in the chronicity index<br>-Inclusion of active vascular lesions (lupus vasculopathy/vasculitis) in the classification schema or activity index<br>- Additional definition regarding how many neutrophils is required in a glomerulus to meet criteria for including a glomerulus in the "Neutrophils/karyorrhexis" score<br>- Clarification as to whether or not fibrous crescents also count towards total glomerulosclerosis score<br>- Clarification as to whether globally sclerotic glomeruli with features of global fibrous crescents are to be scored in the GS score or FC score or both<br>- Chronicity index may underestimate chronicity. For example a case with 100% global glomerulosclerosis and 100% IFTA may be scored as 9 out of 12 if GS glomeruli don't look like old fibrous crescents.<br>- Guidance on how should significant lesions such as crescents, necrosis, or wireloop deposits be scored when present only in the IF or EM tissues? As well significant chronic lesions present only in these tissues<br>- While I feel this is clear; emphasis and clarification regarding the denominator for % of glomerular involvement by any lesions. There seems to be some renal pathologist that score the % active lesions based on total non-globally sclerotic glomeruli. My understanding of the ISN/RPS classification and my approach to active lesions in any GN is to score % active lesions based on total # of glomeruli (non-sclerotic + GS). I think clarrifying this point would be helpful; as I feel some may be greatly overestimate this activity indexes in their reports<br>- There is no clinically meaningful difference between class I and II lupus nephritis; can this category be merged into a single "mesangial limited lupus nephritis" class?

58	Just some explanation about activity index for membranous lupus nephritis
59	I do not think anything needs to be changed with the glomerular classification of lupus nephritis.
60	1. Difference between Class 3 and class 4 is slightly subjective and not qualitative (both indicate proliferative active lupus nephritis), unlike the other classes (1, 2 and 5). It can be merged into a single class X with subtypes Xa and Xb.<br>2. Incorporation of a podocyte-driven separate class (with predominant proteinuria) in the LN classification which can include the newer described subtypes like - a)MCD-like and b) collapsing-like<br>3. Incorporation of vascular findings in the main LN classification, particularly, TMA.<br>4. Acknowledgment of an ANCA-like proliferative variant with prominent fibrinoid necrosis and muted subendothelial deposits on EM
61	still confusion about how to count chronic lesions (sclerosis, fibrous crescents) in assigning Class; significance of adhesions. Needs better definitions of vascular lesions particularly TMA, lupus vasculopathy-- does any TMA preclude lupus vasculopathy; does any deposit preclude TMA? To me, not logical that fibrous crescents can have cellularity (up to 25% w/ 2018 defn)
62	specify the global number of glomeruli with activity lesions and global number of glomeruli with chronicity
63	Regarding Class III + V and Class IV + V, EM is necessary to identify supepithelial deposits. It is quite difficult to distinguish subepithelial deposits from supendothelial deposits by IF. Therefore, the lupus nephritis classification should be revised that EM is necessary for this classification, not just an option.
64	Evidence basis for recommendations. AI and CI are not evidence based.
65	Better definition of endocapillary hypercellularity. Meaning of inflammation in areas of interstitial fibrosis vs non-fibrotic interstitium. Inclusion of vascular lesions.
66	Clarifying the significance of the MPGN pattern of injury regarding activity vs. chronicity
67	More subclasses in class 3 and 4: vasculitic features VS non vaaculitic
68	Replace with evidence based lesion scoring to include tubulointerstitial and vascular lesions
69	Add a scoring system for arterial and arteriolar vascular lesions
70	I think the A, A/C and C should be reintroduced. It helped get an overview across with the final class.
71	Including better definition of vascular lesions. Incorporation of EM in select scenarios
72	It is very glomerular-centric and might be improved by addition of vascular lesions (e.g., TMA). Also measures might be taken to clarify definitions and thresholds for the lesions comprising AI and CI to reduce inter-observer variability.
73	Clarification on the following: the proportion of membranous changes to add a Class V, the pertinence of including vascular changes into the classification (such as thrombotic microangiopathy or vascular inmmune deposits) and the clinical significance of reporting membranoproliferative changes.
74	Even when clinically active, the activity indices are usually in the moderate range. I have rarely reported activity index more than 16
75	The definitions of endocapillary hypercellularity are imprecise.<br>Some leukocyte infiltration in the mesangium appears to be active, even though it does not reduce the capillary lumen.Blood vessel lesions warrant proper classification and inclusion as criteria.<br>The characteristics of interstitial inflammation should be assessed for their significance, including the presence of fibrosis and tubulitis, in accordance with the thresholds proposed in Kdigo. Should tubular atrophy be scored separately from interstitial fibrosis?
76	Vascular change description could be helpful.
77	Clarification for Class III and IV especially if only sclerosing lesion present and no active lesion, should these cases be included in these categories or not. There are discrepancies among pathologists (at least in my country) on this matter.Another issue is definition on Class V whether it should be in diffuse pattern or not. Some people just add in membranous component if the features of membranous are not diffuse especially if there are concomitant Class III/IV.
78	Please continue perfecting the morphological definitions. The 2018 paper was really useful in that sense.
79	consider interstitium and extraglomerular vasculature
80	An official synoptic report template which can be used in a standardized fashion and integrated into laboratory information systems to facilitate discrete data field and dat extraction in a fashion to aggregate and compare between centers.

81	Is determining the distinction between global & segmental glomerulosclerosis secondary to lupus nephritis versus other causes matter given that the chronic change present (irregardless of whether due to lupus or not) is irreversible and deciphering the cause of the glomerulosclerosis is subjective and unreliable.
82	if podocytopathy alone is considered lupus nephritis
83	Addressing in a diagnostic classification the manifestations of lupus nephritis outside of the glomerular patterns.
84	Challenging question. Simplify if possible.
85	simplify the criteria for proliferation and activity
86	1. Remove "fibrous crescents" from the chronicity index (can be lumped with segmental scarring)<br>2. Try to equalize the activity/chronicity indices numerically (24 vs 12), or introduce a percentage system, or a tiered mild/moderate/severe descriptor, or other.<br>3. Promote use of descriptive pattern of injury-based terminology in the topline/main diagnosis.
87	interstitial inflammation definitions
88	Data-driven approach with thresholds reflecting prognosis
89	Need to have a better understanding of the molecular/cellular underpinnings of lupus nephritis, verified in clinical context, in order to improve morphologic classifications.
90	As long as activity and chronicity indices are reported, the distinction between class III and IV does not seem to add to any clinical meaning/implication for treatment plans.<br>The inclusion of global an segmental glomerulosclerosis in the same chronicity score (total glomerulosclerosis) may cause confusion/misinterpretation. Differentiating between segmental glomerulosclerosis and fibrous crescent may occasionally be difficult. Segmental glomerulosclerosis may be the result of ongoing/active podocyte loss that may require immunosuppression.<br>The classification still relies exclusively on morphology.<br>In the future aa more pathogenesis associated classification may become feasible.<br>When commercially available, exostocin positivity in class V may need to be reported.
91	To make clear distinction between lupus nephritis class IV and IV+V
92	Specify whether percentages of glomeruli include or exclude counting globally sclerosed glomeruli.<br>Clarify the difference between necrosis and karyorrhexis.
93	I always get confused about the notion of whether sclerotic glomeruli (either segmentally or globally) can be "attributed to the lupus activity". I'm not sure how one can definitively determine if sclerosis in a glomerulus is from lupus nephritis or from other causes. Most of the time we obviously assume it's due to lupus activity, but how can we definitively state that?
94	The combined score ranges of both NIH indexes needs to be adjusted to be more relevant / predictive (based on comprehensive validation studies). We need an "activity" index for LMN (validated before proposed for use).
95	we need stronger language for minimum adequacy criteria than "As a general rule, 10 seems to be the appropriate number of glomeruli for evaluation."
96	I think that the best way is disscusion during the meeting or by phone with clinician
97	Some comments about the re- biopsy
98	Stop changing it
99	The modified activity/chronicity scoring is very tedious and also seems somewhat arbitrary - that is, the entire score can be changed by finding an additional globally sclerotic glomerulus on a section, which can change all the percentages. It might be helpful to have an online program (like the Banff scoring schema) that will categorize the cases - but that won't change the fact that it is tedious (and arbitrary)! I work in a very high volume practice so I end up getting a lot of lupus cases.
100	Quantitation for parameter can increase reproducibility. Like, set up a cut off for PMN infiltration and karyorrhexis. How many PMN or karyorrhexis in one glomerulus can be count in?<br>Endocapillary hypercellularity: how many cells in one glomerular capillary loop can be considered as endocapillary hypercellularity? Sometimes, there are a few leukocytes in one loop which was not expanded. Are those can be counted as endocapillary hypercellularity?

101	Tubulointerstitial and vascular involvement is missing<br>Poor interobserver variability in scoring<br>Activity/chronicity scores not entirely predictive of outcomes
102	I think that MPGN pattern and crescentic/extracapillary pattern should be separated in different classes. Currently cases with MPGN pattern in >50% and cases with crescents in >50% both are class IV. But the latter on is likely much more aggressive and might benefit from another therapy than "MPGN-like" cases.
103	Simplicity, specificity of scoring, situation of 30% chronic + 30% active (is that class 3 or 4), adding in standardized assessment of vascular lesions, defining neutrophils in glomeruli more specifically, defining exactly how to score (looking at 1 slide vs all slides
104	It is OK, the changes are quite minor and cosmetic
105	Do not separate class I and class II.<br>Any crescent should be considered important, even less than 10% of the circumference.<br>More than 50% of the crescent with a fibrous component should be included in the chronicity index.
106	Adding vascular lesion on classification<br>Adding a cut point to the number of cells to consider endocapilllary hypercellularity
107	Contribution of EM although classification seems to be LM based. Relevance of 'lupus podocytopathy'
108	Clarification regarding the assessment of globally sclerotic glomeruli. This is a problem without an apparent solution.
109	Vasculitis scoring
110	More vascular pathology.<br>More definitions of chronic glomerular lesions.
111	Many of the minimum criteria for active and chronic lesions should be defined. Necrotizing lesions and crescents should not be separate categories (they are the same process just separated points in time). Adhesion, fibrous crescents, segmental sclerosis need to be lumped together. Activity/chronicity should be emphasized over focal/diffuse. Too much confusion by nephrologists when chronic lesions are considered in focal vs diffuse (i.e class III vs IV). An inactive but chronic lupus nephritis should be categorized on it's own somehow (not just class VI with no one ever biopsies since it is so end stage). Minimum criteria for class V need to be better specified.
112	Including the presence and number of crescents; including the presence of acute vascular lesions
113	Simplification. The justification for 2x score for necrosis/crescents/karryorrhexis is not evidence-based
114	Develop a CAP synoptic for lupus nephritis - electronic data entry and capture .

Supplemental Table 4 - Respondents' answers to Question 10 (open text): *'Any other comments?'.*

**Bajema et al., Kidney Int, 2018*

ISN/RPS, International Society of Nephrology/Renal Pathology Society

	**Any other comments?**
1	Useful system. One of the classifications most accepted and understood by clinical colleagues.
2	It would be desirable to incorporate IF and EM results into the classification, since these are used for diagnosis in many if not most institutions. They might be considered optional, to accomodate those centers without the ability to do EM or IF.
3	I find the ISN/RPS system to provide a useful framework for communicating extent and severity of LN
4	In my view all you need is:<br>1. Class/Activity: mesangial, focal, diffuse. Can add membranous if present (either alone or with focal/diffuse)<br>2. Chronicity: a simple chronicity score (0-10) like the Mayo chronicity score.
5	in my opinion karyorrhexis is not always associated with neutrophilic granulocytes
6	Work to do......
7	The following issue was raised in the 2018 publication and needs to be addressed again: "Incorporating globally sclerotic glomeruli that resulted from non-lupus injury in a chronicity index would overestimate the lupus nephritis chronicity and severity, but may still correlate with outcome. This issue should be addressed by phase 2 studies."
8	I don't think the nephrologists even read deep into the comments. They seem to remain using the 2004 Classification. Not sure if this is due to Education of the new Classification or just clinical utility.
9	All classifications fall down when let loose on the world - for example, the Oxford classification of IgA nephropathy
10	It would be very helpful to have pictures of all glomerular lesions (endocapillary hypercellularity, fibrocellular crescent, karyorrhexis, etc.) at the minimum threshold that the lesions can be designated as such.
11	It can be difficult to explain to a nephrologist that a class V lupus nephriits has "0" as an activity score in the NIH index -- as a floridly nephrotic patient clearly has an active disease. This comes up now that the recommendation is to provide activity and chronicity for all biopsies (in the past I had simply not computed the activity for pure LN5)
12	micrscopic photography of many examples for each morphological picture
13	The working group has done a tremendous and useful job in further improving the application of the classification. Thank them for this.
14	You may want to do the same survey among nephrologists to see the difference in the perceived usefulness of the classification. Happy to help disseminate among the 6k members of our newsletter.
15	eventual adding of a vascular scoring
16	Even before 2018, we had always included a comment in our reports detailing the activity and chronicity scoring per Austin and Balow. The huge benefit from the 2018 revision was to clarify and standardize the scoring, which was a little vague in the original papers from the 80's. Our clinicians, particularly the rheumatologists, expect to hear the activity and chronicity scores in our prelim and final reports and incorporate these numbers into their treatment decisions.
17	The activity index should be restructured so that fibrinoid necrosis and cellular crescents are combined as 1 parameter. It is not possible to reach an activity index of 24 points. I think it is really difficult to reach 18 points.
18	IF and EM should be a part of the main classification update and not LM alone, as sometimes, EM findings can be discordant with LM findings.
19	maybe specify the distribution of deposits by IF in interstitium and vessels and the presence of vasculitis by LM
20	Clarity on best way to classify 'grey zone' situations like - looks like Class II with segmental holes and scant capillary wall IF.<br>Also - any recommendations to the clinician on how much a jump or reduction in activity/chronicity scores is significant. Additional features on EM of significance- HCQ induced myelin figures. Been seeing alot of these.
21	Evidence-based confirmation/possible modification of the weighting and thresholds of the lesions comprising AI and CI would make these indices more valuable.
22	If clinicians work closely with pathologists (which they do at our institution) they will have a good understanding of morphology and pathology findings. We review each biopsy with the clinicians and management decisions are

	highly impacted by the pathologist's input. This has been the practice at our institution for a long time, however, adoption of a fully digital workflow in 2016 made this operationally much easier.
23	The classification still relies exclusively on morphology.<br>In the future aa more pathogenesis associated classification may become feasible.<br>When commercially available, exostocin positivity in class V may need to be reported.
24	Sometimes histomorphological changes like hypercellularity or thickening of GBM are not that prominent but glomerular involvement is diffuse. DIF study also doesn't show fullhouse antibody depostion. Such cases are often difficult to interpret.
25	Really like the simplification of the 2018 classification!
26	Is it possible to have another meeting to discuss this survey? These surveys are very useful but people who are not a part of the working group rarely get to see the results or discuss their contributions. Thank you for all your work on this!
27	Whay about Electron Microscopy?
28	Is there real value in continually rehashing the ISN scoring system, which at its core does not show much improvement over WHO system and it doesn't include minimal change, collapsing GN, wire loop-only GN, and vascular disease that occurs in SLE patients. Older nephrologists ask for simpler WHO classification.
29	What about lupus associated podocytopathy?
30	Commenting on hypercellular vs hyaline deposit type activity?
31	Consider including other alterations associated with SLE, not lupus nephritis itself, as "other lesions associated with SLE", e.g. lupus podocytopathy, residual chronic changes without lupus activity, vasculitis and/or lupus vasculopathy.
32	I would like to see wider involvement in evidence gathering and classification development
33	The 2003 ISN/RPS Classification of LN was a monumental step. I was glad to see that the 2018 version only made minor changes, removed the LN IV-S vs. LN IV-G, and re-instated activity and chronicity indices. It was smart to make small but significant changes rather than anything more radical. I think we are currently in a good place with the 2018 version.
34	Would love to participate in any group to modify the current classification since I think there are many practical concerns.
